# Social Media and Men’s Health: Separating Science from Speculation in Andrology

**DOI:** 10.1186/s12610-025-00275-0

**Published:** 2025-07-10

**Authors:** Michael George, Vaibhav Modgil, Ian Pearce, Theodora Stasinou

**Affiliations:** 1 Manchester Royal Infirmary, Manchester University NHS Foundation Trust, Oxford Road, Manchester, M13 9WL UK; 2 Manchester Andrology Research Collaborative, Manchester, UK

**Keywords:** Social media, Communication, Men’s health, Urology, Andrology, Myths, Misinformation, Health promotion, Education, Médias sociaux, Communication, Santé masculine, Urologie, Andrologie, Mythes, Désinformation, Promotion de la Santé, Education

## Abstract

**Background:**

Social media has rapidly evolved into a primary source of health-related information. For men with concerns regarding their sexual health or fertility, consulting the internet is often felt to be an accessible first-step in exploring their symptoms. Medical misinformation and over-generalisation online remain a significant concern amongst medical professionals, particularly in the current social media era of virality and sensationalism. This narrative review aims to explore the growing intersection between social media and andrology, with insights into the current medical media landscape and the opportunity to enhance health promotion.

**Methods:**

The search strategy applied core search terms central to the scope of the review (such as “social media” and “andrology”) in combination with condition-specific terms (such as “erectile dysfunction” or “testosterone”) using Boolean operators to identify relevant literature within PubMed, which served as the primary electronic database, as well as online websites and social media platforms.

**Results:**

Social media consumption is on the rise, with users consuming over two hours daily on average worldwide. Amongst posts, there has been a shift towards highly engaging short-form video content, which is particularly popular with young adults. Social media algorithms reinforce consumption patterns by continuously delivering content based on user engagement: the more an individual interacts with a topic or creator, the more related content appears, creating a self-sustaining feedback loop; this principle underpins the concept of virality and risks the rapid dissemination of inaccuracies online. Key men’s health topics such as infertility, erectile dysfunction, testosterone supplementation and semen retention are popular amongst social media posts, amassing millions-to-billions of views depending on the platform. Amongst these posts, there is a high degree of misinformation. Clinicians should be confident in navigating the possible challenges associated with social media.

**Conclusion:**

Whilst the growing presence of urologists online may have the potential to counter health-related misinformation, there are barriers inherent to social media platforms that limit the reach and impact of expert voices at present. To leverage the opportunity that social media offers, urologists are required to modernise their approach to communication in order to ensure that accurate health information is both accessible and engaging.

**Supplementary Information:**

The online version contains supplementary material available at 10.1186/s12610-025-00275-0.

## Résumé

**Contexte:**

Les médias sociaux sont rapidement devenus une source principale d’information sur la santé. Pour les hommes qui s’inquiètent de leur santé sexuelle ou de leur fertilité, la consultation d’Internet est souvent considérée comme une première étape accessible pour explorer leurs symptômes. La désinformation médicale et la généralisation excessive en ligne restent une préoccupation majeure chez les professionnels de la santé, en particulier à l’ère actuelle de la viralité et du sensationnalisme sur les réseaux sociaux. Cette revue narrative vise à explorer l’intersection croissante entre les médias sociaux et l’andrologie, avec un aperçu du paysage médiatique médical actuel et la possibilité d’améliorer la promotion de la santé. La stratégie de recherche a appliqué des termes de recherche de base essentiels à l’objectif de la revue (tels que « médias sociaux» et « andrologie») en combinaison avec des termes spécifiques à une affection (tels que « dysfonction érectile» ou « testostérone») à l’aide d’opérateurs booléens pour identifier la littérature pertinente dans PubMed, qui a servi de base de données électronique principale, ainsi que dans les sites Web en ligne et les plateformes de médias sociaux.

****Résultats**:**

La consommation des médias sociaux est en hausse, les utilisateurs consommant en moyenne plus de deux heures par jour dans le monde entier. Parmi les publications, il y a eu une évolution vers un contenu vidéo de courte durée très attrayant, qui est particulièrement populaire auprès des jeunes adultes. Les algorithmes des médias sociaux renforcent les modes de consommation en diffusant en permanence du contenu basé sur l’engagement des utilisateurs: plus un individu interagit avec un sujet ou un créateur, plus le contenu connexe apparaît, créant ainsi une boucle de rétroaction auto-entretenue. Ce principe sous-tend la notion de viralité et risque de provoquer une diffusion rapide d’inexactitudes en ligne. Les principaux sujets de santé masculine tels que l’infertilité, la dysfonction érectile, la supplémentation en testostérone et la rétention de sperme sont populaires parmi les publications sur les réseaux sociaux, accumulant des millions à des milliards de vues selon la plateforme. Parmi ces messages, il y a un haut degré de désinformation. Les cliniciens doivent être confiants pour naviguer dans les défis possibles associés aux médias sociaux.

Bien que la présence croissante des urologues en ligne puisse avoir le potentiel de contrer la désinformation liée à la santé, il existe des obstacles inhérents aux plateformes de médias sociaux qui limitent la portée et l’impact des voix d’experts à l’heure actuelle. Pour tirer parti de l’opportunité qu’offrent les médias sociaux, les andrologues/urologues sont tenus de moderniser leur approche de la communication afin de s’assurer que des informations de santé précises sont à la fois accessibles et attrayantes.

## Introduction

Patients have commonly turned to the internet to make sense of their symptoms before consulting a doctor. Their motivations are varied: some wish to better inform discussions with healthcare providers, whilst others seek reassurance or even self-diagnosis which risks non-presentation or catastrophising minor symptoms. In the United States of America, 72% of internet-users report to consult the internet for health information [1], with over one-third seeking a definitive self-diagnosis [2].

In previous years, men with andrological concerns might have consulted online search engines, such as Google, seeking advice. However, today, this search may take place on social media platforms such as YouTube, TikTok, Instagram and X (formerly Twitter). The way people seek health information has shifted considerably; a key and recent example of this shift in this practice came during the COVID-19 pandemic, at which time social media was recognised as a primary source of health information [3–5].

It is widely accepted that online search engines house a wealth of valuable information. Whilst the phenomenon of *‘Dr Google’* may be seen to represent a challenge in navigating patients’ expectations and concerns during present-day consultations [6], it could be argued to support mutual understanding for patients consulting their physician [7]. Despite the unpredictable flaws associated with searching the internet, such search engines generally favour reputable sources with high volume traffic, offering a degree of stability in the websites accessed. Whilst misinformation certainly exists in traditional online search engines, reputable medical sites still hold significant visibility, representing a readily accessible resource, particularly to those aged over 55 years old [8]. *But where, then, do younger patients go for health advice?*

Social media, which began in the early 2000s as a means for individuals to connect with one-another [9], has rapidly evolved into a dominant force for driving health-related conversations and shaping public perception. Today, social media is not just a space for entertainment, but it is a primary news outlet and a leading source of health information for individuals under the age of 40 years-old [8]. It is estimated that 72% of adults turn to social media as a source of health information, with greater engagement amongst young adults [10]; furthermore, approximately 68% of adolescents report consuming sexual health information online [11]. Platforms such as YouTube, TikTok, Instagram and X allow for instantaneous dissemination of medical content, ranging from educational material published by healthcare professionals to viral trends promoting unverified claims, with users incentivised to compete for attention using bite-sized engaging content. Users are empowered and encouraged to share their own material, as well as interact with the content of others through likes, shares and comments; with this engagement, social media algorithms ensure the delivery of related content. This accessibility fosters an opportunity for education but also risks the rapid and widespread dissemination of medical misinformation; the accuracy of health-related content online, relating to various medical specialties, has come under scrutiny with suggestion that platforms such as YouTube [12], Instagram [13] and TikTok [13] cannot be recommended to patients as reliable resources.

Andrology, an inherently sensitive urological sub-specialty, is particularly at risk by this shift in patient practice. Issues related to male reproductive and sexual health often carry stigma, leading many men to delay seeking medical advice or avoid it altogether; this is particularly the case for those already reluctant to seek medical assistance. Concerns about fertility, erectile dysfunction or testosterone levels can feel particularly personal, making an anonymous online search or scroll through social media an accessible first step. However, the reliability of the information returned is unpredictable and poses potentially significant consequences ranging from undue anxiety to inappropriate self-treatment with unregulated and unsupervised supplementation and/or medication. An up-to-date evaluation of the accuracy of online medical information is crucial for identifying the risks and challenges currently confronting the field of andrology.

This narrative review aims to explore the evolving intersection between social media and andrology by consolidating and summarising the existing literature, offering insights into the current medical social media landscape, the profession’s shared responsibility to combat misinformation and the opportunity to modernise patient communication.

## Methods

This narrative literature review utilised a search strategy to identify relevant literature within PubMed, which served as the primary electronic database, as well as online websites and social media platforms between 05/03/2025–01/04/2025. Core search terms central to the scope of the review (such as “social media”, “men’s health”, “urology”, “andrology”) were combined with condition-specific terms (such as “erectile dysfunction” or “testosterone”) using Boolean operators to capture relevant publications; additional discriminatory terms of relevance were used in conjunction with these to facilitate further sub-section analyses, for example the addition of the term “medico-legal” to explore the guidance for clinicians on this sub-topic. The reference list of identified articles were manually screened to consider related publications. Whilst formal inclusion–exclusion criteria were not applied, publications were required to be English-written and accessible online to be eligible for review; naturally, articles that were not relevant to the scope of this review or not accessible were therefore not considered. Articles and references were recorded and stored respectively using EndNote [14].

### Today’s social media landscape

**Fig. 1:**
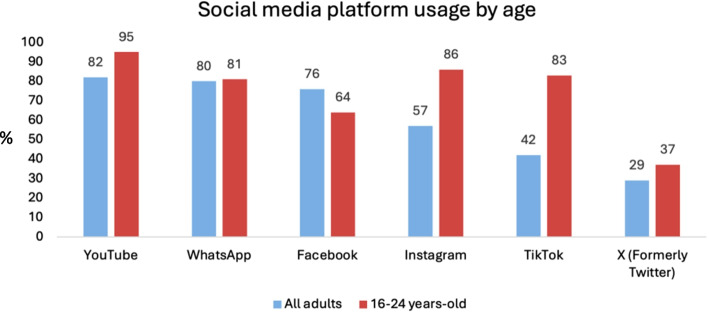
Social media platform engagement amongst all adults and young adults in the UK, expressed as a percentage of individuals accessing the internet [15]. Graphical illustration demonstrating social media platform usage amongst all adults (blue) and young adults (red) in the UK, generated using data from Ofcom’s national annual survey of social media use in the UK [15]. Amongst all adults, YouTube represents the most popular platform followed by WhatsApp, Facebook, Instagram and TikTok sequentially. In those aged 16–24 years, YouTube remains the most popular platform, however Instagram and TikTok are the second and third most popular respectively

Social media has become integral to modern-day life, with 89% of United Kingdom internet users actively engaging with various platforms [15]. On a global scale, users spend an average of over two hours per day on social media [16]. Amongst UK adults, YouTube represents the most popular platform followed by WhatsApp, Facebook, Instagram and TikTok [15]; however, in those aged between 16–24 years-old, YouTube, Instagram and TikTok dominate [15], reflecting a broader shift towards short-form video content. Platform popularity in the UK, as determined by Ofcom’s 2024 annual survey, has been visualised in Fig. 1 [15]. This format, particularly when executed effectively by content creators, is highly engaging and often encourages prolonged viewing. Social media algorithms reinforce consumption patterns by continuously serving content based on user engagement: the more an individual interacts with a topic or creator, the more related content appears, creating a self-sustaining feedback loop; this mechanism underpins the concept of virality. Whilst efforts have been made to combat misinformation – such as the introduction of X’s ‘community note’ function, which allows users to flag and append misleading posts – the sheer volume of content makes comprehensive regulation nearly impossible. Moreover, social media platforms incentivise views and engagement, which can promote a culture of sensationalism. Whilst some creators pursue this with the production of high-quality content, others may push ethical boundaries to capture attention. As more people turn to social media for news and information, ensuring the accuracy of online content is becoming increasingly important [16]. However, more than one in seven UK adults reportedly “never or rarely” verify the credibility of the information they consume [15]. In the context of healthcare, misinformation can have serious consequences, posing a significant concern for the medical profession.

The impact of social media influencers has surged in recent years, particularly following the COVID-19 pandemic which reshaped both consumer behaviour and marketing strategies [17]. Influencers have become key players in the digital space, capable of shaping public perception; many individuals leverage their platforms to drive discussions on topics either of interest to them, their industry partners or to capitalise on topics of particular popularity, which often encompasses health and wellbeing. Increasingly, clinicians and medical institutions are establishing a presence on social media, with some embracing the role of a ‘medfluencer’. The growing digital footprint of urologists presents a valuable opportunity to educate the public, counter misinformation, direct individuals to appropriate services and share professional insights. However, as is the case for all influencers, medfluencers operate within a system that prioritises engagement and viral appeal. Unlike their lay counterparts, clinicians must balance their professional responsibilities with the need to capture attention; this may potentially make it more challenging for evidence-based, educational content to gain traction in an environment that could be argued to, at times, reward sensationalism over substance.

### Trending andrology topics online

#### Overview

Men’s health is an important topic that has gained significant traction on social media with widening reach. In the andrological online space, key themes searched online include infertility, erectile dysfunction, testosterone supplementation, Peyronie’s disease, premature ejaculation and semen retention.

A recent review spanning several popular men’s health topics on TikTok and X revealed that the top 40 videos on each platform collectively amassed over two billion and three million views respectively [13]. Whilst the majority of these posts were determined to be educational in nature, only 17.3% and 15.5% were posted by healthcare professionals on TikTok and Instagram respectively, with physicians accounting for just 12.9% and 10.3% respectively [13]. It is generally accepted that the accuracy of health information shared online tends to decline as topics become more complex. In the posts analysed, overall content accuracy was poor, however physicians were found to provide more reliable information compared to non-professional content creators [13]. Highlighting their concerns regarding misinformation, *Dubin *et al*.* made an example of semen retention which the authors found represented the most popular men’s health topic on social media, despite being surrounded by controversy [13]. Notably, identified posts overwhelmingly endorsed the practice despite limited supportive evidence and negative implications demonstrated by rigorous research [18–21]; research in favour of semen retention has been hindered by small sample sizes, perceived methodological flaws and retraction of previously published work [18, 19]. Coincidentally, this topic was unique in lacking any physician representation amongst the most viral posts identified [13].

#### Erectile dysfunction

Erectile dysfunction represents a core andrological concern, posing significant physical and psychosocial consequences, that draws significant online traffic returning over 30 million results on searching Google [22]; Russo et al*.* delineated trends in online searches within the topic of erectile dysfunction [23]. Fode et al*.* undertook a review of the top 100 YouTube videos on the topic, available in English, which amassed nearly 36 million views collectively [24]. Despite the vast majority of these videos discussing medical material, approximately 40% of authors were of no discernible medical background [24]. On analysis, the authors found that videos produced by medical professionals/institutions and those free from commercial advertising achieved significantly higher levels of information accuracy [24]; the converse was true for those produced by lay individuals and/or those with a commercial interest. A similar review of 116 videos published on YouTube by Quirós et al*.* classified videos as either scientifically evidence-based (38%) or non-scientifically evidence-based (62%) on expert review [25]. The former were observed to more commonly discuss aetiology, pathophysiology, clinical features and conventional treatment options, whilst the latter more often discussed natural/alternative remedies and personal accounts [25]. Interestingly, videos determined to be non-scientifically evidence-based achieved significantly more views than their evidence-based counter-parts [25].

Related studies have performed focussed assessment specific to the management of erectile dysfunction. Pezone et al. evaluated YouTube videos discussing phosphodiesterase-5-inhibitors in erectile dysfunction, with the authors reviewing 229 videos discussing either one or a combination of sildenafil, tadalafil, vardenafil and avanafil [26]. Overall, the content discussed within these videos was found to be challenging for patients to understand and apply, with an average understandability and actionability score of 55% and 0% respectively using the Patient Education Materials Assessment Tool (PEMAT) [26], in which the threshold value of 70% typically determines satisfactory performance in each domain [27]. Furthermore, the accuracy of information discussed was also unsatisfactory, with a median DISCERN score of 29.5 (interquartile range [IQR] 18–41) and median misinformation scale scores ranging between 1–2 in each domain [26]. The authors expressed their concern regarding the degree of misinformation online and its potential clinical implications [26]. Similarly, Capece et al*.* reviewed YouTube content on the topic of penile prosthesis in the management of erectile dysfunction [28]. At the time of study conduction, the search term “penile prosthesis” yielded nearly 9000 videos, with the first 100 videos returned undergoing evaluation [28]. The included videos amassed over 10 million views collectively [28]. The videos exhibited a mean PEMAT understandability score of 57.7 (standard deviation [SD] 19.2%) and median actionability score of 0% (IQR 0–33%), with content published by hospital organisations achieving significantly improved understandability score compared to that published by medical professionals or private companies [28]. The median DISCERN score was 26 (IQR 21–30), with the accuracy of 32 and 57 of the 100 videos assessed to be ‘poor’ or ‘very poor’ respectively [28]. Sellke et al*.* evaluated the quality of information relating to the treatment of erectile dysfunction posted on Reddit, a forum-based social media platform, encompassing a breadth of treatment options including sexual behaviour, lifestyle modifications, medical therapies, talking therapies and supplementation [29]. The authors found that just 24.4% of the management options discussed were in line with American Urological Association guidelines [29]; fewer than half of positive testimonies posted by users endorsed a treatment that was guideline-supported [29]. Reddy et al*.* and Pattenden et al*.* appraised information on erectile dysfunction management found on websites identified through Google searching, rather than on social media, finding the quality and readability of the information to be similarly poor [30, 31].

#### Peyronie’s disease

Peyronie’s disease is another andrological condition that has gained attention on social media, with a review of X (formerly Twitter) posts demonstrating growing discussion online [32]. A review by Baydilli et al*.* assessed the quality of educational YouTube videos on the topic, with content initially screened by two independent urologists and further appraised using the DISCERN scale and Global Quality Score [33]. Of the 267 most-viewed videos analysed, nearly half contained inaccurate information [33]. Concerningly, many of these misleading videos promoted traction devices and homeopathic remedies with unfounded supportive evidence [33]. The more accurate videos were more commonly produced by physicians, educational institutions and professional organisations, whereas the majority of inaccurate content came from independent users and for-profit companies [33].

#### Male factor infertility

Male factor infertility often carries particular stigma, meaning its discussion online requires a careful, non-judgmental approach to ensure men wishing to conceive receive accurate, evidence-based advice. A review by Zaila et al*.* examined the reliability of online articles shared on social media, finding that, once again, nearly half contained inaccurate or misleading information [34]; the authors concluded that, whilst social media can increase awareness, misinformation is widespread and often fuelled by a culture of sensationalism [34]. Similarly, Ku et al*.* assessed YouTube videos on male infertility using a questionnaire based on American Urological Association guidelines [35]. They found that 90% of the sampled videos had little educational value, with the 10% of greater yield created by healthcare professionals or organizations [35]. Research suggests that peer support and personal testimonials can be helpful for patients [36, 37]; however, whilst positive experiences may offer hope, they can also create unrealistic expectations, whereas negative stories may contribute to pessimism or worry [38].

#### Premature ejaculation

Premature ejaculation risks significant distress, further amplified by its accompanying stigma. A single-day snapshot survey by Bernstein et al. appraised related content on TikTok, finding 80% of videos were unreliable [39]. The authors observed that physicians representation was significant greater in the reliable sub-group, whilst those published by self-identified patients or for-profit organisations were associated with unreliable content [39]. A review conducted by Gul et al*.* found that 70% of videos published on YouTube were deemed to reliably discuss premature ejaculation [40], suggesting a marked difference between the platforms. Both Bernstein et al. and Gul et al*.* did, however, once again conclude that non-reliable videos were more commonly published by medical advertisements, for-profit organisations and non-qualified individuals [39, 40].

#### Phimosis

Phimosis, characterised by the inability to partially or completely retract the foreskin over the glans, risks genital pain and sexual dysfunction. Recognising the pitfalls observed in YouTube’s portrayal of other key men’s health topics, Cilio et al*.* conducted an analysis of content on the subject of phimosis [41]. The authors sampled 60 eligible videos presented on the first page of results on searching the website [41]. Sharing likeness in methodology to previously discussed studies, these videos underwent assessment using the DISCERN, PEMAT and misinformation indexes once again. Healthcare providers authored 75% of videos [41]. The median understandability score was 42.9% (IQR 34.5–58.9), actionability score 50.0% (IQR 25.0–56.) and misinformation score 2.8 (IQR 1.6–3.6) [41]. The authors concluded that the quality of phimosis information available on YouTube is poor and requires significant improvement before it can be recommended as a source for information to patients [41], compounding the findings of related research. Ho et al. reported the contrasting viewpoint of phimosis-related information available via non-social media webpages on searching Google, finding information challenging to read for lay persons and of poor overall accuracy (DISCERN 41.5 [IQR 34–51]) [42]; interestingly, the authors did, however, find that higher ranked pages with increased visibility achieved significantly greater accuracy metrics compared to lower ranked pages [42].

#### Testicular pain

Testicular pain is a common presentation that prompts men to consult their urologist, with a broad spectrum on possible underlying causes. Systematic clinical assessment and timely intervention is essential, especially in cases of clinical acuity such as testicular torsion. Social media may offer a valuable resource in educating patients on these differential diagnoses and appropriate medical response; however, misinformation online risks harmful outcomes. Melchionna et al*.* conducted an analysis of 117 videos on the topic identified on searching YouTube, finding the content to be unsatisfactory in terms of its understandability, actionability and accuracy overall [43]. Bai et al*.* conducted a similar cross-sectional study, sampling the first 100 results on searching YouTube using the term “testicular torsion” and subsequently analysing 66 videos after application of inclusion–exclusion criteria [44]. The overall quality of content was poor amongst videos, with information published by physicians significantly more accurate than that published by individual users [44]. Bai et al*.* concluded that the degree of misinformation observed may pose significant health risks to those consuming it, emphasising the need to improve the quality of information available to patients online [44]. A related and important aspect of patient education within urological care centres upon testicular self-examination, supporting the early detection of pathology such as testicular cancer; Selvi et al*.* conducted a review of informative videos published on YouTube, finding the content to be of high quality but challenging to locate for the lay person unfamiliar with medical jargon [45].

### Guidance to clinicians in the social media era

A thorough understanding of the current social media landscape, including its opportunities and challenges, is essential for modern-day clinical practice. This extends beyond posting and consuming content, encompassing a deeper awareness of patient expectations and digital behaviours. As healthcare becomes increasingly integrated with the online space, clinicians must develop the skills to navigate social media with ethical and professional integrity. Doing so enables them to harness its benefits whilst mitigating potential risks, ultimately promoting patient-centred care.

In the United Kingdom, the General Medical Council (GMC) provides clear guidance on professional conduct for clinicians using social media [46, 47]. Additionally, medical indemnity organisations such as the Medical Defence Union (MDU) offer medico-legal recommendations [48], whilst professional bodies like the European Association of Urology (EAU) provide specialty-specific advice [49]. These guidelines advocate for professionalism and integrity, urging clinicians to uphold the same standards online as they would in public or the workplace. The core principles of safe social media use – honesty, accountability, integrity, confidentiality and accuracy – are already familiar to many urologists. However, as social media continues to evolve, clinicians must remain vigilant about how these principles intersect with daily practice. Juliebø-Jones et al. highlight key considerations, including the potential for patients to record clinical encounters (with or without disclosure) and to publicly discuss clinicians'performance online [50]; their work also offers practical strategies to support responsible social media engagement among urologists [50].

Beyond its application in health promotion and education, social media serves as a valuable platform for professional development with scope to connect with colleagues, foster new relationships, acquire knowledge, share research and technological advances and recruit to studies [51–54].

## Discussion

This narrative review systematically explored existing literature and online resources to map the current landscape of men's health on social media. Andrological symptoms are often perceived to be sensitive and/or embarrassing by patients, leading many men to seek information online instead of consulting a specialist. With social media becoming a key health resource, the spread of misinformation poses serious risks. This review demonstrated widespread misinformation and oversimplifications across key men's health topics, with less-accurate content often gaining more visibility and engagement.

As outlined within the condition-specific discussions, the accuracy of health information was found to vary between social media platforms, with TikTok videos, compared to YouTube, found to be less reliable and more likely promote unsupervised and/or alternative supplements on the whole [55]. Barbar et al*.,* further underscored the impact of short-form video content on men’s health, noting that TikTok videos garnered significantly more views, likes and followers than YouTube videos, despite being less than one-tenth of the duration on average [55]. The degree of misinformation reported amongst TikTok posts raises notable concern, particularly in view of the shift towards this format of content consumption amongst young adults and the platform’s increased reach and engagement metrics in this field compared to YouTube.

It is worthwhile acknowledging that there is a wealth of valuable evidence-based information available to patients online, including patient information resources published by reputable institutions and organisations such as the British Association of Urological Surgeons [56] and the European Association of Urology [57]. These resources are accessible to patients and free-of-charge but require patient awareness of their existence and an understanding of how to access them. Furthermore, written information, particularly in the format of lengthy prose, may be perceived to be less accessible or enticing by patients compared to the audio-visual content available on social media.

Social media is a powerful tool for driving health-related conversations and shaping public perception. However, significant concerns remain within the medical profession regarding the prevalence of health misinformation and oversimplification online, both of which can heavily influence patients'engagement with healthcare systems. Yet, amidst the noise, there is an opportunity. As social media continues to shape public perspectives, clinicians must actively engage, educate and challenge misleading narratives to ensure that accurate, evidence-based discussions on men’s health prevail in an era where digital influence is more powerful than ever. Recognising this opportunity, Sawchuk et al*.* published their experience introducing a novel social media campaign as a means to increase public awareness and education on the crucial urological topic of testicular torsion [58]; the campaign reached over 26,000 unique Instagram accounts and 14,500 unique Facebook accounts during the study period, achieving over 80,000 views on paid advertisement and demonstrating improved awareness amongst those surveyed [58].

Many published studies rightly advocate for urologists to utilise social media to combat misinformation and debunk health-related myths, a stance we strongly support. However, little attention is given to the broader challenges inherent to these platforms. Social media thrives on sensationalism and spectacle, and frankly, conventional methods of delivering informative material often lack the appeal required to compete with viral trends. For messages to be received, voices must be heard. Addressing the misinformation epidemic should be the collective responsibility of content creators, consumers, healthcare professionals, governmental organisations and social media platforms. Social media platforms may assist with tighter regulation of users and fact-checking of content, perhaps with scope to integrate technological advances such as artificial intelligence to lighten the burden; additionally, enforcement of platforms’ terms of service, as well as governmental regulations, may support this. However, with regards to urological practice, this article serves as a call to action: an innovative approach is needed to modernise the way healthcare information is delivered to patients, ensuring that accurate, evidence-based content cuts through the noise of unfounded claims in order to effectively promotes men’s health.

Whilst the current evidence base is in its infancy, few studies within the existing literature have explored the manner in which social media interfaces with andrological conditions, as well as core urological conditions such as prostate or testicular cancer [59–61]; amongst these studies, there is a preponderance to examine the accuracy of health-related information on YouTube, with a sparsity of research into TikTok and Instagram which represent key sources of information today. With future studies continuing to comprehensively probe the topic of men’s health on social media, it may be possible to delineate trends in the degree of misinformation online. There is also an unmet need to better understand the manner in which andrological patients engage with social media to clarify real-world exposure to such misinformation, for example by establishing tendencies to search symptoms on social media platforms or fact-check the information they consume; this would better determine the scale of the challenge faced, allowing for combative measures to be tailored for maximal effect.

## Study Limitations

Despite growing interest in the interface of social media with men’s health, there remains a sparsity of studies investigating each topic in depth, reflecting both the breadth of social media platforms and andrological topics. This gap is more evident for platforms with an emerging healthcare influence such as TikTok and Instagram, further compounded by changes in content format, popularity and user practices which challenge long-term analysis.

This review met its objective of synthesising the existing literature to provide an overview of andrology-related content on social media. However, it is subject to limitations inherent to narrative reviews; despite the use of a structured search strategy, the lack of a formalised systematic search protocol with strict inclusion and exclusion criteria introduces the possibility of selection bias and subjectivity in the interpretation of findings. In future, a systematic review could address these limitations; however, such an approach would likely necessitate a narrower focus, perhaps confined to a single platform or a specific andrological condition, and thus would not be suited to the broad, exploratory aim of the present review.

Future research is desirable to build upon the foundations of the existing literature. It would be beneficial to better understand the behaviours of UK patients engaging with health information on social media, delineating their tendency to search for health information online and fact-check the information they consume. It would also be interesting to evaluate for temporal trends in information accuracy, particularly in view of the increasing evidence-base over the previous years.

## Conclusion

Social media has become a key source of health information for many individuals. Misinformation and generalised advice pose significant risks to andrological care. Whilst the growing presence of urologists online may have the potential to counter these issues, there are barriers inherent to social media platforms that limit the reach and impact of expert voices at present. To leverage the opportunity that social media offers, we as urologists are required to modernise our approach to communication in order to ensure that accurate health information is both accessible and engaging to patients.

## Supplementary Information

Supplementary Material 1: Supplementary Fig. 1. Info-graphic summarising key findings and discussion points. Social media is central to modern day life, with 9 in 10 adults in the UK actively engaging with platforms and global users consuming over 2h daily. YouTube is the most common app amongst all adults in the United Kingdom; however, there has been a shift towards Instagram and TikTok amongst young adults. As health information becomes increasingly accessible on social media, there is a risk of widespread misinformation which is compounded by the fact that 1 in 7 adults in the UK reportedly “never or rarely” verify the credibility of the information they consume. Men’s health topics are popular amongst social media posts, with widespread misinformation and over-generalisation available. It is essential that urologists are competent in maximising the potential of social media to enhance patient education and care, whilst also addressing misinformation where it arises.

## Data Availability

Not applicable.

## References

[1] Fox S, Duggan M. Health Online 2013 - Pew Research Centre. Accessible online: https://www.pewinternet.org/wp-content/uploads/sites/9/media/Files/Reports/PIP_HealthOnline.pdf; 2013 15/01/2013.

[2] Kuehn BM. More than one-third of US individuals use the Internet to self-diagnose. JAMA. 2013;309(8):756–7. 23443421 10.1001/jama.2013.629

[3] Zhong Y, Liu W, Lee TY, Zhao H, Ji J. Risk perception, knowledge, information sources and emotional states among COVID-19 patients in Wuhan. China Nurs Outlook. 2021;69(1):13–21. 32980153 10.1016/j.outlook.2020.08.005 PMC7442898

[4] Sallam M, Dababseh D, Yaseen A, Al-Haidar A, Taim D, Eid H, et al. COVID-19 misinformation: Mere harmless delusions or much more? A knowledge and attitude cross-sectional study among the general public residing in Jordan. PLoS ONE. 2020;15(12): e0243264. 33270783 10.1371/journal.pone.0243264 PMC7714217

[5] Neely S, Eldredge C, Sanders R. Health Information Seeking Behaviors on Social Media During the COVID-19 Pandemic Among American Social Networking Site Users: Survey Study. J Med Internet Res. 2021;23(6): e29802. 34043526 10.2196/29802 PMC8202660

[6] Kłak A, Gawińska E, Samoliński B, Raciborski F. Dr Google as the source of health information – the results of pilot qualitative study. Polish Annals of Medicine. 2017;24(2):188–93.

[7] Van Riel N, Auwerx K, Debbaut P, Van Hees S, Schoenmakers B. The effect of Dr Google on doctor-patient encounters in primary care: a quantitative, observational, cross-sectional study. BJGP Open. 2017;1(2):bjgpopen17X100833. 10.3399/bjgpopen17X100833 PMC6169945 30564661

[8] Fice H. How is social media changing andrology? In: Robaire B, Chan P, editors. Handbook of Andrology. Third Edition ed. Available online: https://andrologysociety.org/andrology-handbook/: The American Society of Andrology; 2023.

[9] Ortiz-Ospina E. The rise of social media. Accessible online: https://ourworldindata.org/rise-of-social-media: Our World in Data; 2019 [

[10] Erlandsson LC, Guijo MS, Quintana-Alonso R. Decoding patterns: the crucial role of social media in health information consumption and user dynamics among the general population. Journal of Public Health. 2024.

[11] González-Ortega E, Vicario-Molina I, Martínez JL, Orgaz B. The Internet as a Source of Sexual Information in a Sample of Spanish Adolescents: Associations with Sexual Behavior. Sexuality Research and Social Policy. 2015;12(4):290–300.

[12] Collà Ruvolo C, Morra S, Di Bello F, Cilio S, Fraia A, Polverino F, et al. A systematic review assessing the reliability of studies focusing on urological content on YouTube. Minerva Urol Nephrol. 2025;77(2):192–201. 40298344 10.23736/S2724-6051.24.05994-9

[13] Dubin JM, Aguiar JA, Lin JS, Greenberg DR, Keeter MK, Fantus RJ, et al. The broad reach and inaccuracy of men’s health information on social media: analysis of TikTok and Instagram. Int J Impot Res. 2024;36(3):256–60. 36402921 10.1038/s41443-022-00645-6 PMC9676765

[14] Team TE. EndNote. EndNote X9 ed. Philadelphia, PA: Clarivate; 2013.

[15] Ofcom. Adult’s Media Use and Attitudes Report. Accessible online: https://www.ofcom.org.uk/siteassets/resources/documents/research-and-data/media-literacy-research/adults/adults-media-use-and-attitudes-2024/adults-media-use-and-attitudes-report-2024.pdf?v=321395; 2024.

[16] GWI. Social: Behind the Screen. Accessible online: https://www.gwi.com/reports/social: Global Web Index; 2023.

[17] Ng IKS, Thong C, Tan LF, Teo DB. The rise of medical influencers: The pros and the cons. Journal of the Royal College of Physicians of Edinburgh. 2024;54(3):231–5. 38867442 10.1177/14782715241261736

[18] Exton MS, Krüger TH, Bursch N, Haake P, Knapp W, Schedlowski M, et al. Endocrine response to masturbation-induced orgasm in healthy men following a 3-week sexual abstinence. World J Urol. 2001;19(5):377–82. 11760788 10.1007/s003450100222

[19] Jiang M, Xin J, Zou Q, Shen JW. A research on the relationship between ejaculation and serum testosterone level in men. J Zhejiang Univ Sci. 2003;4(2):236–40. 12659241 10.1631/jzus.2003.0236

[20] Elzanaty S, Malm J, Giwercman A. Duration of sexual abstinence: epididymal and accessory sex gland secretions and their relationship to sperm motility. Hum Reprod. 2005;20(1):221–5. 15550495 10.1093/humrep/deh586

[21] Levitas E, Lunenfeld E, Weiss N, Friger M, Har-Vardi I, Koifman A, et al. Relationship between the duration of sexual abstinence and semen quality: analysis of 9,489 semen samples. Fertil Steril. 2005;83(6):1680–6. 15950636 10.1016/j.fertnstert.2004.12.045

[22] Russo GI, Asmundo MG, Durukan E, Fode M. Quality and benefits of the erectile dysfunction information on websites, social-media, and applications. Int J Impot Res. 2024;36(7):688–92. 37369784 10.1038/s41443-023-00725-1

[23] Russo GI, di Mauro M, Cocci A, Cacciamani G, Cimino S, Serefoglu EC, et al. Consulting “Dr Google” for sexual dysfunction: a contemporary worldwide trend analysis. Int J Impot Res. 2020;32(4):455–61. 31591474 10.1038/s41443-019-0203-2

[24] Fode M, Nolsøe AB, Jacobsen FM, Russo GI, Østergren PB, Jensen CFS, et al. Quality of Information in YouTube Videos on Erectile Dysfunction. Sex Med. 2020;8(3):408–13. 32593674 10.1016/j.esxm.2020.05.007 PMC7471071

[25] Mazuecos Quirós J, Pedraza Sánchez JP, Lozano Blasco JM, Baena Villamarín C, Lendínez Cano G, Medina López RA. Is English information about erectile dysfunction on YouTube based on scientific evidence? Int J Urol. 2020;27(10):939–40. 32662122 10.1111/iju.14310

[26] Pezone G, Collà Ruvolo C, Cilio S, Fraia A, Di Mauro E, Califano G, et al. The spreading information of YouTube videos on Phosphodiesterase 5 inhibitors: a worrisome picture from one of the most consulted internet source. Int J Impot Res. 2024;36(7):747–54. 37865715 10.1038/s41443-023-00762-w

[27] Shoemaker SJ, Wolf MS, Brach C. Development of the Patient Education Materials Assessment Tool (PEMAT): a new measure of understandability and actionability for print and audiovisual patient information. Patient Educ Couns. 2014;96(3):395–403. 24973195 10.1016/j.pec.2014.05.027 PMC5085258

[28] Capece M, Di Giovanni A, Cirigliano L, Napolitano L, La Rocca R, Creta M, et al. YouTube as a source of information on penile prosthesis. Andrologia. 2022;54(1): e14246. 34519075 10.1111/and.14246

[29] Sellke N, Jesse E, Callegari M, Muncey W, Harris D, Edwins R, et al. Is Reddit a reliable source for information on erectile dysfunction treatment? Int J Impot Res. 2023;35(5):484–9. 35597799 10.1038/s41443-022-00586-0 PMC9123614

[30] Reddy RV, Golan R, Loloi J, Diaz P, Saltzman RG, Watane A, et al. Assessing the quality and readability of online content on shock wave therapy for erectile dysfunction. Andrologia. 2022;54(11): e14607. 36240784 10.1111/and.14607

[31] Pattenden TA, Raleigh RA, Pattenden ER, Thangasamy IA. Quality and readability of online patient information on treatment for erectile dysfunction. BJUI Compass. 2021;2(6):412–8. 35474701 10.1002/bco2.87 PMC8988690

[32] Balasubramanian A, Yu J, Lipshultz LI, Hotaling JM, Pastuszak AW. #Peyronies: An Analysis of Online Twitter Discussion of Peyronie’s Disease. Urol Pract. 2020;7(1):75–81. 37317414 10.1097/UPJ.0000000000000057

[33] Baydilli N, Selvi I. Is social media reliable as a source of information on Peyronie’s disease treatment? Int J Impot Res. 2022;34(3):295–301. 34172941 10.1038/s41443-021-00454-3

[34] Zaila KE, Osadchiy V, Shahinyan RH, Mills JN, Eleswarapu SV. Social Media Sensationalism in the Male Infertility Space: A Mixed Methodology Analysis. World J Mens Health. 2020;38(4):591–8. 32378368 10.5534/wjmh.200009 PMC7502321

[35] Ku S, Balasubramanian A, Yu J, Srivatsav A, Gondokusumo J, Tatem AJ, et al. A systematic evaluation of youtube as an information source for male infertility. Int J Impot Res. 2021;33(6):611–5. 32541795 10.1038/s41443-020-0322-9 PMC8445813

[36] Blakemore JK, Bayer AH, Smith MB, Grifo JA. Infertility influencers: an analysis of information and influence in the fertility webspace. J Assist Reprod Genet. 2020;37(6):1371–8. 32382959 10.1007/s10815-020-01799-2 PMC7205373

[37] Quaas AM. Social media in ART-#power or #peril? J Assist Reprod Genet. 2020;37(6):1311–2. 32468332 10.1007/s10815-020-01831-5 PMC7311600

[38] Kelly-Hedrick M, Grunberg PH, Brochu F, Zelkowitz P. “It’s Totally Okay to Be Sad, but Never Lose Hope”: Content Analysis of Infertility-Related Videos on YouTube in Relation to Viewer Preferences. J Med Internet Res. 2018;20(5): e10199. 29792296 10.2196/10199 PMC5990861

[39] Bernstein A, Zhu M, Loloi J, Babar M, Winokur N, Wysocki M, et al. TikTok as a source of information regarding premature ejaculation: a qualitative assessment. Sexual Medicine. 2023;11(2). 10.1093/sexmed/qfac020 PMC9978577 36910705

[40] Gul M, Diri MA. YouTube as a Source of Information About Premature Ejaculation Treatment. J Sex Med. 2019;16(11):1734–40. 31522984 10.1016/j.jsxm.2019.08.008

[41] Cilio S, Collà Ruvolo C, Turco C, Creta M, Capece M, La Rocca R, et al. Analysis of quality information provided by "Dr. YouTube(TM)" on Phimosis. Int J Impot Res. 2023;35(4):398–403. 10.1038/s41443-022-00557-5 PMC8942804 35332276

[42] Ho YT, Salinas J, Ayeni F, Ranasinghe S, Arianayagam M, Canagasingham B, et al. Quality and Readability of Google Searches on Phimosis and Paraphimosis Management. Trends in Urology & Men’s Health. 2025;16(1): e12002.

[43] Melchionna A, Collà Ruvolo C, Capece M, La Rocca R, Celentano G, Califano G, et al. Testicular pain and youtube™: are uploaded videos a reliable source to get information? Int J Impot Res. 2023;35(2):140–6. 35136203 10.1038/s41443-022-00536-w

[44] Bai G, Pan X, Zhao T, Chen X, Liu G, Fu W. Quality Assessment of YouTube Videos as an Information Source for Testicular Torsion. Front Public Health. 2022;10: 905609. 35664123 10.3389/fpubh.2022.905609 PMC9157819

[45] Selvi I, Baydilli N, Akinsal EC. Can YouTube English Videos Be Recommended as an Accurate Source for Learning About Testicular Self-examination? Urology. 2020;145:181–9. 32791289 10.1016/j.urology.2020.06.082

[46] GMC. Using social media as a medical professional. General Medical Council; 2024.

[47] GMC. Good medical practice. General Medical Council; 2024.

[48] MDU. Using social media. Accessible online: https://www.themdu.com/guidance-and-advice/guides/guide-to-social-media2024

[49] Yuen-Chun Teoh J, Bhatt NR, Cucchiara V, Garcia Rojo E, Gauhar V, Mercader C, et al. Best Practice in Using Social Media: The European Association of Urology Position Statement. Eur Urol. 2025;87(2):104–7. 39472201 10.1016/j.eururo.2024.10.018

[50] Juliebø-Jones P, Gauhar V, Keller EX, Coninck V, Talyshinskii A, Sierra A, et al. Social media and urology: The good, the bad and the ugly. Urologia. 2024;91(4):659–64. 39212156 10.1177/03915603241273885 PMC11481405

[51] Salem J, Borgmann H, Murphy DG. Integrating Social Media into Urologic Health care: What Can We Learn from Other Disciplines? Curr Urol Rep. 2016;17(2):13. 26757907 10.1007/s11934-015-0570-2

[52] Saade K, Shelton T, Ernst M. The Use of Social Media for Medical Education Within Urology: a Journey Still in Progress. Curr Urol Rep. 2021;22(12):57. 34913134 10.1007/s11934-021-01077-3 PMC8674028

[53] de Graaf BC, Gerritse MBE, Michiels KCJ, Kluivers KB, van de Belt TH. Social media recruitment of participants in a female stress urinary incontinence trial: A feasibility study. Eur J Obstet Gynecol Reprod Biol. 2024;299:253–7. 38908036 10.1016/j.ejogrb.2024.06.028

[54] Farah OK, Wang CN, Chung DE. The role of facebook support groups for women with benign urologic conditions. Neurourol Urodyn. 2023;42(8):1795–801. 37705338 10.1002/nau.25283

[55] Babar M, Loloi J, Patel RD, Singh S, Azhar U, Maria P, et al. Cross-sectional and comparative analysis of videos on erectile dysfunction treatment on YouTube and TikTok. Andrologia. 2022;54(5): e14392. 35122283 10.1111/and.14392

[56] BAUS. Information Leaflets Accessible online: https://www.baus.org.uk/patients/information_leaflets2024

[57] EAU. EAU Patient Accessible online: https://patients.uroweb.org/.2025

[58] Sawchuk T, Metcalfe P. Teste Talk: A trial social media campaign to improve awareness of testicular torsion. Can Urol Assoc J. 2023;17(5):E110–5. 36758185 10.5489/cuaj.8135 PMC10132373

[59] Xue X, Yang X, Xu W, Liu G, Xie Y, Ji Z. TikTok as an Information Hodgepodge: Evaluation of the Quality and Reliability of Genitourinary Cancers Related Content. Front Oncol. 2022;12: 789956. 35242704 10.3389/fonc.2022.789956 PMC8885733

[60] Xu AJ, Taylor J, Gao T, Mihalcea R, Perez-Rosas V, Loeb S. TikTok and prostate cancer: misinformation and quality of information using validated questionnaires. BJU Int. 2021;128(4):435–7. 33811424 10.1111/bju.15403

[61] Di Bello F, Collà Ruvolo C, Cilio S, La Rocca R, Capece M, Creta M, et al. Testicular cancer and YouTube: What do you expect from a social media platform? Int J Urol. 2022;29(7):685–91. 35318754 10.1111/iju.14871

