# Supplementary material for: Social Media and Men’s Health: Separating Science from Speculation in Andrology
Source: Basic Clin Androl. 2025 Jul 10;35:28. doi: 10.1186/s12610-025-00275-0 (PMC12247255; doi:10.1186/s12610-025-00275-0)
Supplement: Supplementary file 1 — Supplementary Material 1: Supplementary Fig. 1. Info-graphic summarising key findings and discussion points. Social media is central to modern day life, with 9 in 10 adults in the UK actively engaging with platforms and global users consuming over 2h daily. YouTube is the most common app amongst all adults in the United Kingdom; however, there has been a shift towards Instagram and TikTok amongst young adults. As health information becomes increasingly accessible on social media, there is a risk of widespread misinformation which is compounded by the fact that 1 in 7 adults in the UK reportedly “never or rarely” verify the credibility of the information they consume. Men’s health topics are popular amongst social media posts, with widespread misinformation and over-generalisation available. It is essential that urologists are competent in maximising the potential of social media to enhance patient education and care, whilst also addressing misinformation where it arises. [file 12610_2025_275_MOESM1_ESM.docx]

**Supplementary materials**

**
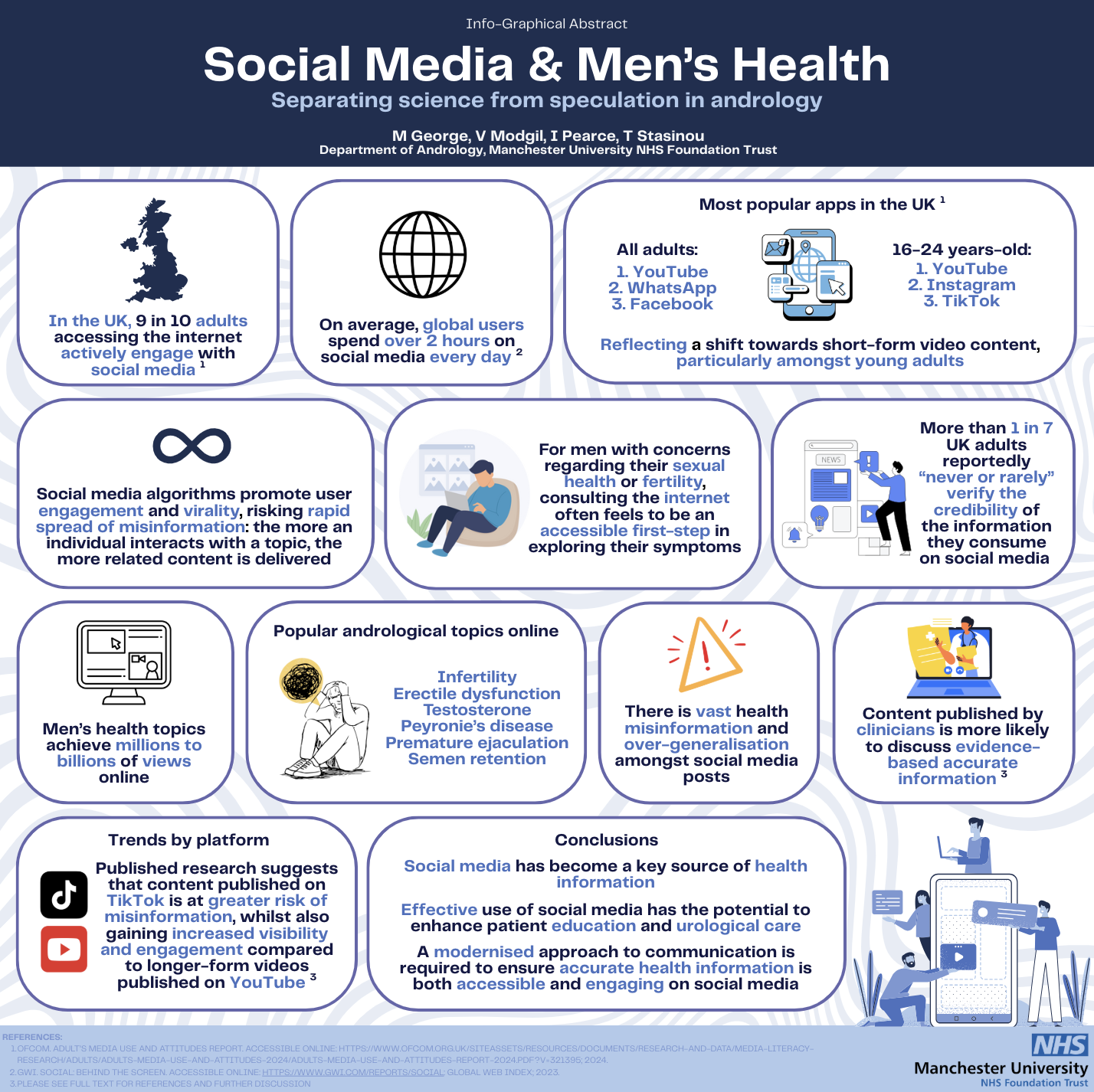
**

**Supplementary Figure 1: Info-graphic summarising key findings and discussion points.** Social media is central to modern day life, with 9 in 10 adults in the UK actively engaging with platforms and global users consuming over 2 hours daily. YouTube is the most common app amongst all adults in the United Kingdom; however, there has been a shift towards Instagram and TikTok amongst young adults. As health information becomes increasingly accessible on social media, there is a risk of widespread misinformation which is compounded by the fact that 1 in 7 adults in the UK reportedly “never or rarely” verify the credibility of the information they consume. Men’s health topics are popular amongst social media posts, with widespread misinformation and over-generalisation available. It is essential that urologists are competent in maximising the potential of social media to enhance patient education and care, whilst also addressing misinformation where it arises.
